# Athlete level, sport-type, and gender influences on training, mental health, and sleep during the early COVID-19 lockdown in Malaysia

**DOI:** 10.3389/fphys.2022.1093965

**Published:** 2023-01-11

**Authors:** Jad Adrian Washif, Lian-Yee Kok, Carl James, Christopher Martyn Beaven, Abdulaziz Farooq, David B. Pyne, Karim Chamari

**Affiliations:** ^1^ Sports Performance Division, Institut Sukan Negara Malaysia (National Sports Institute of Malaysia), Kuala Lumpur, Malaysia; ^2^ Department of Sport and Exercise Science, Faculty of Applied Sciences, Tunku Abdul Rahman University of Management and Technology, Kuala Lumpur, Malaysia; ^3^ Scientific Conditioning Centre, Hong Kong Sports Institute, Hong Kong, Hong Kong SAR, China; ^4^ Division of Health, Engineering Computing and Science, Te Huataki Waiora School of Health, University of Waikato, Tauranga, New Zealand; ^5^ Aspetar, Orthopaedic and Sports Medicine Hospital, FIFA Medical Centre of Excellence, Doha, Qatar; ^6^ Research Institute for Sport and Exercise, University of Canberra, Canberra, ACT, Australia

**Keywords:** elite athlete, injury, mental health, periodisation, recovery, remote training

## Abstract

**Purpose:** We evaluated the extent of changes in training practices, recovery, mental health, and sleep patterns of athletes during the early COVID-19 lockdown in a single country-cohort.

**Methods:** A total of 686 athletes (59% male, 41% female; 9% World Class, 28% International, 29% National, 26% State, 8% Recreational) from 50 sports (45% individual, 55% team) in Malaysia completed an online, survey-based questionnaire study. The questions were related to training practices (including recovery and injury), mental health, and sleep patterns.

**Results:** Relative to pre-lockdown, training intensity (−34%), frequency (−20%, except World-Class), and duration (−24%–59%, especially International/World-Class) were compromised, by the mandated lockdown. During the lockdown, more space/access (69%) and equipment (69%) were available for cardiorespiratory training, than technical and strength; and these resources favoured World-Class athletes. Most athletes trained for general strength/health (88%) and muscular endurance (71%); and some used innovative/digital training tools (World-Class 48% vs. lower classification-levels ≤34%). More World-Class, International, and National athletes performed strength training, plyometrics, and sport-specific technical skills with proper equipment, than State/Recreational athletes. More females (42%) sourced training materials from social media than males (29%). Some athletes (38%) performed injury prevention exercises; 18% had mild injuries (knees 29%, ankles 26%), and 18% received a medical diagnosis (International 31%). Lower-level athletes (e.g., State 44%) disclosed that they were mentally more vulnerable; and felt more anxious (36% vs. higher-levels 14%–21%). Sleep quality and quantity were “normal” (49% for both), “improved” (35% and 27%), and only 16% and 14% (respectively) stated “worsened” sleep.

**Conclusion:** Lockdown compromised training-related practices, especially in lower-level athletes. Athletes are in need of assistance with training, and tools to cope with anxiety that should be tailored to individual country requirements during lockdown situations. In particular, goal-driven (even if it is at home) fitness training, psychological, financial, and lifestyle support can be provided to reduce the difficulties associated with lockdowns. Policies and guidelines that facilitate athletes (of all levels) to train regularly during the lockdown should be developed.

## Introduction

Almost as soon as the Coronavirus disease 2019 (COVID-19) pandemic was declared, daily routines of people worldwide including athletes were disrupted. Mandated lockdowns and other measures associated with varying levels of restriction on movement and social interactions (daily living, occupational and physical activity) were imposed across the globe that negatively impacted factors including mental health, sleep patterns, nutritional intake, and fitness levels (Ammar et al., 2020; Trabelsi et al., 2021; Washif et al., 2022b; Romdhani et al., 2022c). Consequently, athletes, from Recreational to World-Class calibre, were forced to alter their training routines (such as training loads and modalities) due to the imposed constraints (Washif et al., 2022e). These constraints necessitated modified training that was often home-based and streamed on the internet (Ammar et al., 2021; Tjønndal, 2022). This online support was especially evident among elite-level athletes (Washif et al., 2022b).

Lockdowns have been reported to be costly for most nations and created a burden for athletes, both physically and mentally. During the lockdown, some countries (such as Sweden) allowed outdoor activities (Carlander et al., 2022); whereas, in contrast, such activity was strictly prohibited in Malaysia (Washif et al., 2021). Thus, athletes in Malaysia, irrespective of their competition classification level (World-Class, International, National, State, Recreational), were confined to training in their homes (Washif et al., 2021). The environmental conditions of the country (hot and humid) may have imposed additional training challenges on the athletes. In addition to these challenges, not all high-level Malaysian athletes received remote training support from their coaches and trainers at the beginning of lockdown training. Logistical constraints and limited resources delayed the implementation of training assistance and athlete support. A large-scale study involving >12,000 athletes with diverse characteristics from 142 countries reported widespread challenges faced by athletes, including disruptions to training and decreased motivation levels among athletes (Washif et al., 2022b). Following this, a study that focused specifically on a sample of elite Malaysian athletes (*n* = 76) reported negative experiences from mandatory lockdown such as increased mental and emotional stress, fewer nutritional choices, and reduced training motivation (Washif et al., 2021; Washif et al., 2022a). These results are comparable to the conditions faced by South African athletes (Pillay et al., 2020).

Male and female athletes have reported different training experiences during the COVID-19 pandemic. Female athletes are inclined to be involved with online training, while male athletes were more likely to participate in virtual sports competitions (Tjønndal, 2022) in their own homes. Virtual sports are based on simulator platforms (digital technology) or a treadmill/bicycle connected to a computer (with screen monitor) that enables actual and live running/pedaling. The objective is to replicate racing realities, hills, headwinds, drafting effects, and/or even online competition with other athletes. Male athletes also appear to have maintained a higher number of weekly training days and hours, to a greater extent than female athletes (Mon-López et al., 2020). As well as differences between gender, differences between sporting types were identified, with sports requiring specialist facilities such as swimming, shooting, archery, and team sports often unable to carry out technical training at training sites (Washif et al., 2022e). However, some athletes were able to perform specific skills or fitness training such as running for endurance athletes, throwing for shot putters, weight training for weightlifters, and continuance of strength development if possessing training equipment at home (Washif et al., 2022b; Meier et al., 2022; Pagaduan et al., 2022).

Sleep quantity and quality are of paramount importance for recovery and performance of the athletes. Reduced training intensity and fewer training sessions result in circadian interference, which negatively impacts sleep quality (Romdhani et al., 2022b) and could potentially trigger mental health issues (Facer-Childs et al., 2021), such as elevated stress and depression (Facer-Childs et al., 2021; Romdhani et al., 2022b). In this context, high intensity training can promote 1) increased sleep drive contributing to improved sleep (Romdhani et al., 2022b) and 2) activation of endogenous opioid release (inside the brain) resulting in mood elevation and stress reduction (Saanijoki et al., 2018). Unfortunately, mental distress and the athletes’ inability to maintain athletic routines have also been associated with an increased injury risk (Wiese-Bjornstal, 2019; Rampinini et al., 2021b). Uncertainty around the resumption of competitions while in lockdown situation, may worsen mood, reduce motivation, and amplify eventual mental health issues (Facer-Childs et al., 2021).

Although some studies have proposed how the lockdown has affected athletes’ training practices, injury prevalence, sleep disorder occurrences, and mental-health-related challenges during the lockdown, patterns of discrepancies between gender (male vs. female), and sport-type (individual vs. team-based sports) require clarification. Currently, very few studies have investigated differences due to athlete classification levels (Mon-López et al., 2020; Pillay et al., 2020). Higher classification-level athletes (i.e., World-Class, International) would likely have higher needs for training equipment, frequency, facilities and intensity than lower classification-level athletes (e.g., State).

The aim of this study was to evaluate training and recovery practices, injury prevalence, mental perspectives, and sleep patterns of athletes in Malaysia during the early stages of lockdown, including changes in key training variables with references to athlete classification level, sport type, and gender. This information will elucidate the effects of lockdown among athletes in Malaysia to develop appropriate mitigation approaches for future lockdowns, and/or challenging lockdown-like situations. We expected that athletes from higher classification levels (e.g., World Class), and individual-based sports, would better maintain key training variables compared to athletes from other classification levels and sports.

## Materials and methods

### Design

A within-subject, cross-sectional, questionnaire study was conducted 2 months after the country’s announcement of a lockdown, from 17 May–5 July 2020. This study was part of a global survey investigating athletes’ training knowledge, beliefs, and practices during the COVID-19 lockdown (Washif et al., 2022b). Overall data and comparisons for differences according to athlete competition classification level (World-Class, International, National, State, and Recreational), sport-type (individual e.g., athletics, badminton, karate vs. team-based sports e.g., hockey, soccer, rugby), and gender (male vs. female) were made. Sport type comparison excluded parasports given a possible confounding factor in the grouping of para-athletes. Athlete classification was based on their highest competition level e.g., Olympic or world championship representatives or similar caliber athletes were grouped as World-Class; participation at other international-, continental-, regional-, and inter-community competitions as International; participation at national-level competition as National; participation at state-level competition as State; and other non-competitive sports participation, usually for leisure, health, or work-related as Recreational (Washif et al., 2022b; Washif et al., 2022e).

### Respondents

Participant eligibility is described elsewhere (Washif et al., 2022b). Briefly, only athletes aged 18 years old or above, and who had not missed training for 7 days or more due to illness and/or injury during the lockdown, were allowed to participate in the survey. These participants provided informed consent prior to participation. The study was conducted in accordance with the Declaration of Helsinki, and approved by the institutional research committee of Institut Sukan Negara, Malaysia (ISNRP004-21).

### Sample size

Taking into account the rate of sports participation (two-thirds of the population) in Malaysia (www.iyres.gov.my), a response distribution of 50% (which maximises sample size), a Z-score or standard deviation of 2.6 (for a 99% confidence interval), and the margin of error of ∼5%, the sample size requirement of 664 athletes was calculated for the current study. A final sample of 686 consenting athletes was subsequently collected and analysed.

### Questionnaires

**FIGURE 1:**
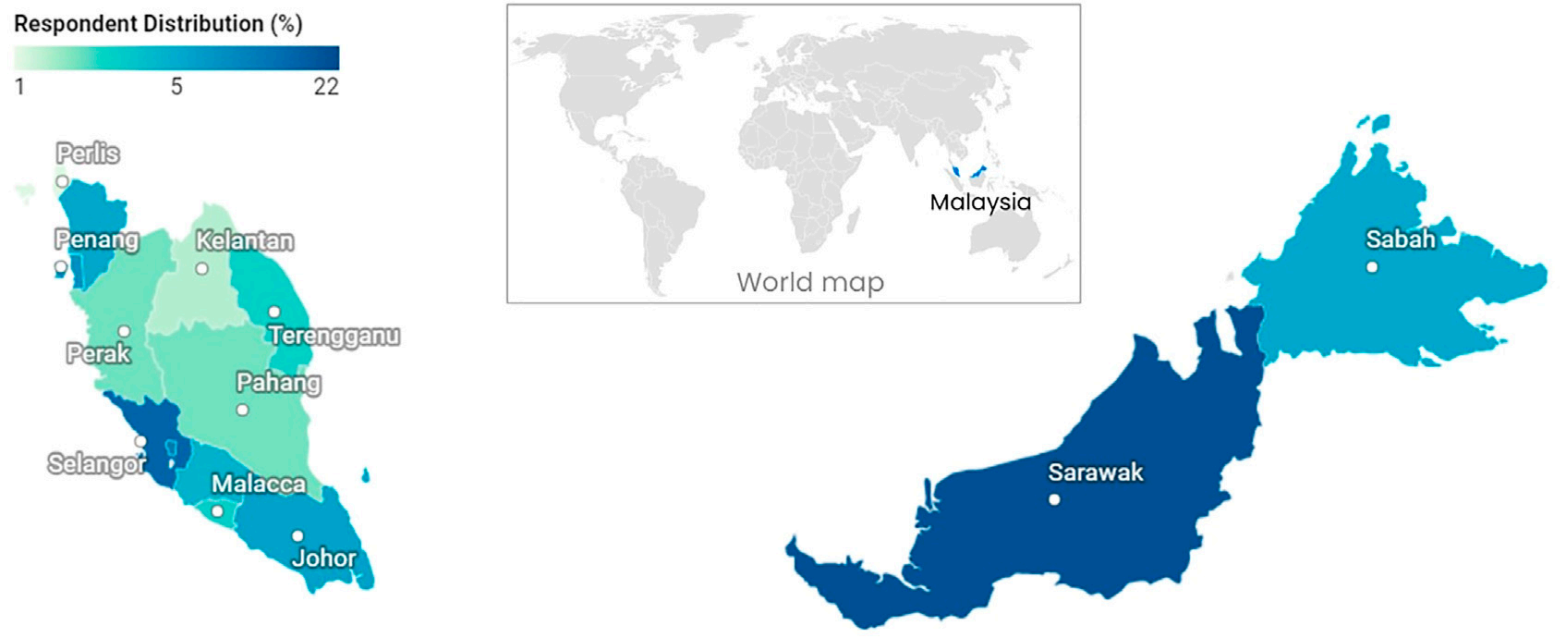
Distribution of respondents based on athletes’ residence in Malaysia.

**FIGURE 2:**
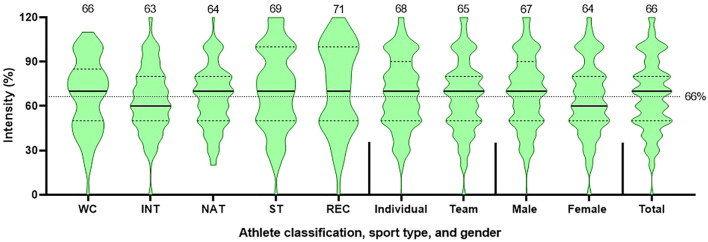
Training intensity depicted by athlete classification, sport-type, gender, and total cohort (*n* = 665). (Question: Do/did you maintain your pre-lockdown intensity for sports specific training (practicing your sport) during the lockdown? Can you estimate how much in percentage? (100% represents the same intensity as before the lockdown). Note: The violin plot includes a 5-point summary, which represents the number in dataset: minimum (lower extreme); 25% percentile (first dashed line/lower quartile); median (black thick line); 75% percentile (second dashed line/third quartile); and maximum (upper extreme). The thin dotted line across all charts/violins represents average intensity.

**FIGURE 3:**
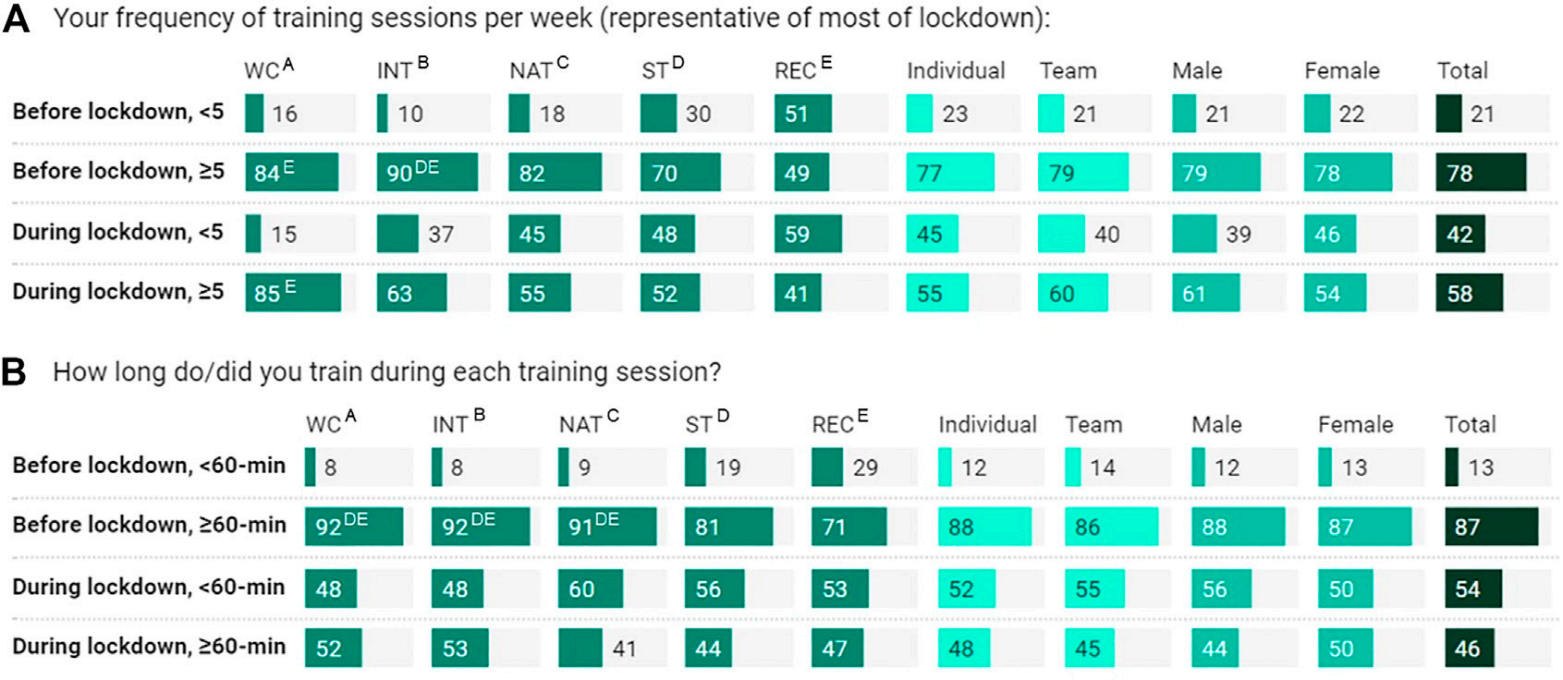
Training frequency (< or ≥ 5 sessions/week; **(A)**) and duration (< or ≥ 60 min/session **(B)**) based on athlete classification, sport-type, gender, and total cohort, before and during lockdown; data are column % of respondents (*n* = 661). Percentage, within athlete classification, sport-type, and gender represents “yes” answer, relative to “no” answer. WC, world-class; INT, international; NAT, national; ST, state; REC, recreational. *Significantly higher (or significant contributor to the relationship); superscript letter represents significantly higher than ^A/B/C/D/E^; all at *p* < .05, based on residuals of >1.96.

**FIGURE 4:**
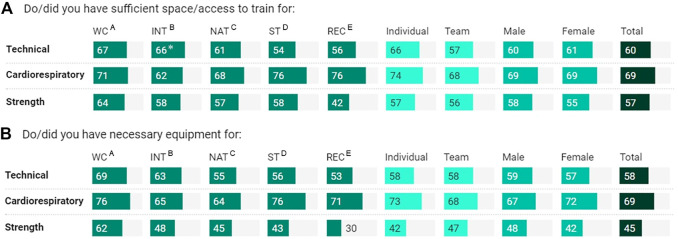
Training space/access **(A)** and equipment **(B)** for technical, cardiorespiratory, and strength by athlete classification, sport-type, gender, and total cohort (*n* = 660). Percentage, within athlete classification, sport-type, and gender, represents “yes” answer, relative to “no” answer. WC, world-class; INT, international; NAT, national; ST, state; REC, recreational. *Significantly higher (or significant contributor to the relationship); superscript letter represents significantly higher than ^A/B/C/D/E^; all at *p* < .05, based on residuals of >1.96.

**FIGURE 5:**
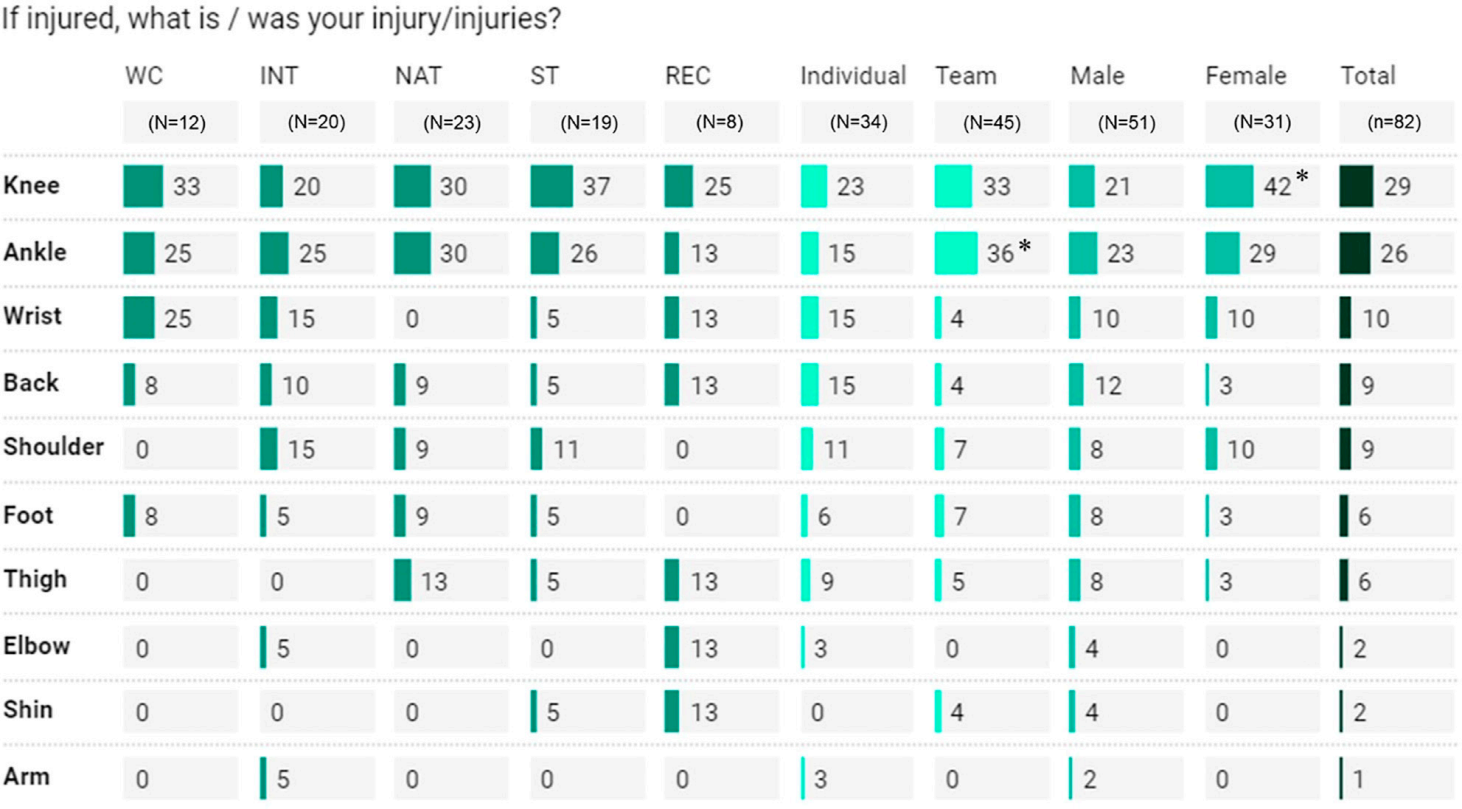
Injury prevalence as shown by muscle areas Percentage, within athlete classification, sport-type, and gender, represents “yes” answer, relative to “no” answer. WC, world-class; INT, international; NAT, national; ST, state; REC, recreational. *Significantly higher at *p* < .05 (within the specific comparative variable).

**FIGURE 6:**
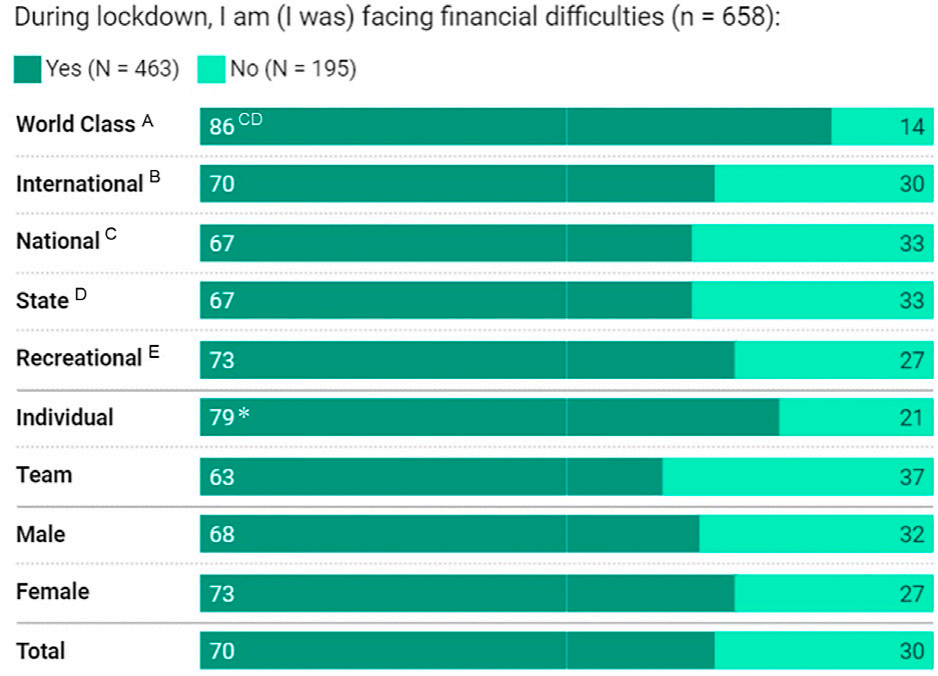
Financial challenges during lockdown based on athlete classification, sport-type, gender, and total cohort; data are presented in percentage. ^CD^Significantly higher than National and State, *significantly higher at *p* < .05 (within the specific comparative variable).

The survey was developed initially by the first (JAW) and last (KC) authors, then reviewed and revised by the wider authorship team, involving >100 researchers from >60 countries. In the present study, questions were partially taken form the global study of Washif et al., 2022b. The socio-demographic section comprised 7 questions; Table 1; Figure 1 (Washif et al., 2022b). The training practice section comprised 11 questions; Table 2, Figures 2, 3, 4 (Washif et al., 2022b). Monitoring, recovery, and injury comprised of 8 questions (Table 3; Figure 5). Psychological responses and financial challenge section comprised 5 questions (Table 4; Figure 6). Sleep habits comprised 4 questions (Table 5). These questions involved: 1) selecting one or more predefined answers; 2) comparing related pre-to during-lockdown effects on training practices; 3) yes or no; and 4) sub-questions including a free-text cell to capture details (Washif et al., 2022b). Responses were carefully screened (e.g., exclusion criteria), cleaned (e.g., removal of duplicates), checked for genuinity, and then converted into standardised codes/numbers to facilitate statistical analysis. Test-retest reliability of the survey was rated as good-excellent (ICCs of >.82).

**TABLE 1:** Socio-demographic characteristics of the respondents in Malaysia during the 2020 COVID-19 lockdown under a national Movement Control Order (*n* = 686).

	Number	Percentage
Gender		
Male	405	59.0
Female	281	41.0
Age groups, years		
18–29	559	81.7
30–39	94	13.8
>40	31	4.5
Missing	2	(−)
Sports experience, years		
≤3	137	20.8
4–8	309	47.0
9–12	166	25.2
>12	46	7.0
Missing	32	(−)
Residence (state/Federal Territory)		
Sarawak	145	21.1
Selangor	121	17.6
Federal Territory	94	13.7
Penang	66	9.6
Johor	46	6.7
Kedah	40	5.8
Sabah	39	5.7
Negeri Sembilan	32	4.7
Malacca	24	3.5
Terengganu	23	3.4
Pahang	18	2.6
Perak	18	2.6
Kelantan	12	1.8
Perlis	8	1.2
Main sports		
Athletics	83	12.0
Field hockey	61	8.8
Lawn bowls	49	7.1
Badminton	46	6.7
Squash	40	5.8
Soccer	39	5.7
Tenpin bowling	38	5.5
Volleyball	22	3.2
Rugby	21	3.0
Archery	20	2.9
Karate	17	2.5
Pencak silat	16	2.3
Boxing	15	2.2
Sepak takraw	15	2.2
Handball	14	2.0
Judo	14	2.0
Petanque	14	2.0
Swimming	13	1.9
Sailing	12	1.7
Weightlifting	12	1.7
Other sports	125	19
Athlete classification level		
World class	64	9.3
International	187	27.3
National	199	29.0
State	180	26.2
Recreational	56	8.2
Sport classification		
Team sports	193	28.1
Precision sports	132	19.2
Power/Technical sports	104	15.2
Racquet sports	89	13.0
Combat sports	83	12.1
Aquatics sports	41	6.0
Endurance sports	23	3.3
Parasports	21	3.1
Number of household members		
1 (I live alone)	29	4.2
2	94	13.8
3	88	12.9
4	147	21.6
5 or more	324	47.5
Missing	4	(−)

**TABLE 2:** Training practices during COVID-19 lockdown in Malaysia by athlete classification, sport-type, gender, and total cohort (percentage of respondents).

	Athlete classification					Sport-type		Gender		
	WC^A^	INT^B^	NAT^C^	ST^D^	REC^E^	Ind	Team	Male	Female	Total
1. What are/were your general purpose(s) of training during the lockdown? *To maintain/develop:*										
…general fitness and health	83	91	84	91	84	87	89	89	86	88
…skills/technique	45	49	53	47	51	48	49	53	45	49
…strength and power	56	68	65	66	65	64	66	67	62	65
…muscular endurance	75	73	72	64	75	68	72	72	68	71
…abdominal strength	53	60	58	43	40	50	55	54	51	53
…aerobic fitness	52	62	61	63	56	64	57	61	60	61
…general flexibility	45	54	48	39	42	44	48	48	44	46
To improve muscle balance	52	62^DE^	56^E^	38	36	48	53	53	48	51
Weight management	55	65	59	55	56	61	57	59	59	59
Other	0	1	1	2	2	2	1	1	1	1
2. Who is prescribing/prescribed the training program during the lockdown?										
Own training program	27	27	38	51^AB^	67^AB^	41	41	39	40	40
Coach or trainer	47	65^DE^	60^E^	47	36	50	58	58^*^	50	55
Combination of above	64^DE^	55^DE^	48^E^	37	20	45	47	45	48	46
External source: YouTube etc.	17	35	36^A^	38^A^	31	35	34	29	42^*^	34
Other	0	0	2	1	4	1	1	1	1	1
3. Do/did you train?										
Alone	77	86	85	96^ABC^	93	85	90	90	86	88
With partners of equal capacity	42^DE^	26	24	16	16	24	24	19	30^A^	24
With other family/friends	23	24^D^	15	11	15	19	15	16	18^A^	17
Others	0	0	1	1	0	0	1	0	1	0
4. Do you/have you been using innovative/modern ways to maintain/improve your fitness levels to adapt to the lockdown conditions?										
Yes	48^BCDE^	28	34	30	30	31	33	34	29	32
No	52	72	67	70	70	69	67	66	71	68
5. What are the type of exercises that you are doing/have been doing consistently (at least twice a week) during lockdown?										
Body-weight based exercises	59	66^D^	57	46	52	56	55	53	59	56
Strength/weightlifting training	48^DE^	35^D^	38^D^	20	18	25	35	30	33	32
Technical skills (sport specific)	41	50	44	39	30	40	44	40	45	43
Technical imitation	34	32	28	23	29	28	27	26	30	28
Cardio training (e.g., HIIT)	63	53	57	74^BC^	66	62	65	64	60	62
Plyometrics	16	45^ADE^	44^ADE^	29	23	31	42	37	36	36
Others	0	1	0	2	7	2	1	2	1	1
6. What are the types of specific training you are/were able to do with the same intensity during the lockdown (very similar to pre-lockdown)?										
Warm up and stretching	84	91	87	81	82	85	87	87	85	86
Strength/weightlifting training	35	30	37	28	22	28	33	29	33	31
Plyometrics	24	43^D^	38	28	24	33	37	36	33	34
Technical skills (sport-specific)	30	46^D^	36	30	27	40	31	35	36	36
Speed training	24	40	32	40	44	30	42	31	40*	36
Speed endurance	21	32	27	37	42	28	34	29	33	31
Long endurance	30	33	36	45	44	37	38	35	39	37
Interval training	41	48	45	57	47	48	51	51	48	49
Change of directions	10	16	15	13	7	9	18	10	16*	13
Others	0	1	0	1	0	0	1	1	0	0

Note: Unless otherwise specified, the number of participants for each question is at least 95% (*n* = 650) of the total cohort; WC, world-class; INT, international; NAT, national; ST, state; REC, recreational; Ind, individual sports; Team—team sports. *significantly higher (*p* < .05) or significant contributor to the relationship (for sport-type and gender); and superscript letters. ^A/B/C/D/E^ (for athlete classification) indicates significance (*p* < .05), based on residuals of >1.96.

**TABLE 3:** Monitoring, recovery, and injury by athlete classification, sport-type, gender, and total cohort, during COVID-19 lockdown in Malaysia (percentage of respondents).

	Athlete classification					Sport-type		Gender		
	WC^A^	INT^B^	NAT^C^	ST^D^	REC^E^	Ind	Team	Male	Female	Total
1. If your coach or trainer is/was in contact with you, is/was this?										
At least once a day	41^D^	40^E^	29	18	19	30	29	30	30	30
At least once a week	37	38	42	35	25^ABCD^	34	39	39	34	37
Two to three times a month	13	11	11	13	11	11	12	10	14	12
Once a month or less	3	5	7	7	2	4	7	6	6	6
They never contacted me	3	4	6	13^A^	6	9	6	8	6	7
Other	3	2	5	14^BC^	38^BC^	12	7	8	11	9
2. Is/was there anybody monitoring your training load and/or wellness during your lockdown training?										
Yes	77^DE^	60	62	44	44	57	56	56	57	57
No	23	40	38	56	56	43	44	44	43	43
3. If Yes, who does/did this?										
Sports scientist	16^CE^	14^CE^	6	7	2	10	8	9	8	9
Fitness coach	45^DE^	27^E^	27^E^	19	7	20	27*	28^*^	21	25
Coach	50^DE^	24	17	9	5	14	17	17	16	17
Other	8	4	5	4	12	7	4	3	9	5
4. If Yes, which tools are used/were used to monitor your training load?										
No tools are/were used	20	23	35^D^	18	27	20	28	28*	21	25
Heart rate monitors	20	14	10	18	14	19	12	14	16	15
Rating of Perceived Exertion	27^CD^	13	9	7	7	15	9	10	13	11
Daily diary	23	21	20	21	14	24	16	20	21	20
Questionnaire(s)	33^BCDE^	16	13	11	4	14	14	13	16	14
GPS	6	10	5	8	13	11	6	9	6	8
Other	6	4	5	7	11	8	5	6	6	6
5. What are the modes of physical recovery that you are using/have been using consistently (at least once a week) during the lockdown?										
Not applicable/inconsistent	20	18	24	23	36^B^	22	23	24	20	23
Ice bath	25	19	19	19	9	17	21	21	16	19
Massage	19	24	16	22	29	18	24	22	20	21
Acupuncture	0	1	2	0	0	2	0	1	1	1
Sauna	9^C^	3	2	3	0	4	3	4	3	3
Stretching	61	69	71	68	57	69	67	65	72	68
Meditation/relaxation	22	18	16	18	20	17	20	17	19	18
Other	0	2	1	3	4	3*	1	1	3	2
6. During your training, do/did you include any injury prevention exercises at least once weekly?										
Yes (*n* = 126)	38	37	36	34	30	34	37	38	32	36
No (*n* = 560)	63	63	64	66	70	66	63	62	68	64
7. Did you sustain any mild injury during the lockdown period?										
Yes (*n* = 23)	22	17	18	17	23	19	18	19	17	18
No (*n* = 103)	78	83	82	83	77	81	82	81	83	82
8. If Yes, did you receive a medical diagnosis from a health care professional?										
Yes (*n* = 23)	21	31^D^	16	12	0	16	19	21	12	18
No (*n* = 103)	79	69	84	88	100	85	81	79	88	82

Note: Unless otherwise specified, the number of participants for each question is at least 95% (*n* = 650) of the total cohort; WC, world-class; INT, international; NAT, national; ST, state; REC, recreational; Ind, individual sports; Team – team sports. *significantly higher (*p* < .05) or significant contributor to the relationship (for sport-type and gender); and superscript letters. ^A/B/C/D/E^ (for athlete classification) indicates significance (*p* < .05), based on residuals of >1.96.

**TABLE 4:** Athletes’ psychological responses by athlete classification, sport-type, gender, and total cohort, during a COVID-19 lockdown in Malaysia (percentage of respondents).

	Athlete classification					Sport-type		Gender		
	WC^A^	INT^B^	NAT^C^	ST^D^	REC^E^	Ind	Team	Male	Female	Total
1. Lockdown can make me mentally vulnerable (mental)										
Strongly agree	6	6	7	11	11	9	8	8	8	8
Agree	25	24	25	44^BC^	40	29	33	30	33	31
Neutral	36^D^	29^D^	34^D^	15	22	24	30	28	26	27
Disagree	17	36	27	23	20	29	25	29	25	27
Strongly disagree	16^B^	4	6	8	6	9*	4	6	8	7
Don’t know	0	1	1	0	2	0	1	0	1	1
2. During lockdown I feel/felt anxious (anxiety)										
Strongly agree	8	4	7	7	4	5	6	5	6	6
Agree	14	21	18	36^ABC^	22	20	26	24	23	23
Neutral	39	27	32	24	33	30	29	28	30	29
Disagree	20	41^AD^	33	24	29	31	30	32	30	31
Strongly disagree	17	6	10	8	9	12*	7	9	9	9
Don’t know	2	2	2	2	4	2	2	2	2	2
3. During the lockdown I am/was constantly scared to get infected by the COVID-19 virus										
Strongly agree	10	14	12	12	18	10	16*	14	11	13
Agree	27	36	31	29	27	28	34	31	31	31
Neutral	35	27	30	24	16	28	26	29	23	27
Disagree	21	14	16	22	20	21*	14	13	24*	18
Strongly disagree	6	7	10	12	16	11	9	10	10	10
Don’t know	2	2	2	1	2	2	1	2	1	2
4. Do you/have you been watch(ing) competitions from your sport or practiced mental training in order to work on (improve) your mental skills/performance?										
Yes	58	62^D^	53	42	53	55	50	56	49	53
No	42	38	47	58	47	45	50	44	51	47

Note: Unless otherwise specified, the number of participants for each question is at least 95% (*n* = 650) of the total cohort; WC, world-class; INT, international; NAT, national; ST, state; REC, recreational; Ind, individual sports; Team—team sports. *significantly higher (*p* < .05) or significant contributor to the relationship (for sport-type and gender); and superscript letters. ^A/B/C/D/E^ (for athlete classification) indicates significance (*p* < .05), based on residuals of >1.96.

**TABLE 5:** Sleep pattern during lockdown in Malaysia based on athlete classification, sport-type, gender, and total cohort (percentage of respondents).

	Athlete classification					Sport-type		Gender		
	WC^A^	INT^B^	NAT^C^	ST^D^	REC^E^	Ind	Team	M	F	Total
1. Usually, pre-lockdown I used to have naps during the day time.										
Yes	59	52	55	71^BC^	78^BC^	69*	55	57	65*	61
No	41	48	46	29	22	31	45	43	35	40
2. Usually, during lockdown I used to have naps during the day time.										
Yes	57	51	53	70^BC^	71	66*	54	57	62	59
No	44	49	47	30	29	34	46	43	38	41
3. Compared to pre-lockdown, during lockdown my average sleep quality is/has been:										
Very much improved	3	9	11	6	2	9	7	7	8	8
Improved	30	25	26	19	38^D^	21	28	26	24	25
Normal	52	52	46	47	49	52	47	51	46	49
Worsened	13	12	13	27^BCE^	7	16	16	14	19	16
Very much worsened	3	2	4	1	4	2	3	2	4	2
4. Compared to pre-lockdown, during lockdown my average sleep quantity is/has been:										
Very much improved	3	9	9	7	9	10	6	8	8	8
Improved	30	27	30	21	33	23	30	27	27	27
Normal	50	54	47	47	47	52	47	51	46	49
Worsened	13	10	12	24^BC^	7	14	15	13	16	14
Very much worsened	5	2	3	0	4	2	3	1	3	2

Note: Unless otherwise specified, the number of participants for each question is at least 95% (*n* = 650) of the total cohort; WC, world class; INT, international; NAT, national; ST, state; REC, recreational; Ind, individual sports; Team—team sports. *significantly higher (*p* < .05) or significant contributor to the relationship (for sport-type and gender); and superscript letters. ^A/B/C/D/E^ (for athlete classification) indicates significance (*p* < .05), based on residuals of >1.96.

### Data collection

The survey was conducted *via* Google Forms, which was disseminated *via* e-mail, personal/group messaging applications (e.g., WhatsApp and Telegram) and social media (e.g., Facebook and Twitter) through networks of the research team. All participants were asked to reflect on their “worst experience” of the lockdown (locally known as Movement Control Order) between March and May 2020.

### Statistical analysis

All data were analysed using SPSS v.26 (IBM, Chicago, Illinois, United States). Data are presented using a variety of appropriate descriptive statistics, including frequencies, percentages, and mean ± standard deviation. Mean scores (where related) of two factors (gender, and sport type) or more than two (athlete classification) comparators were compared using an independent *t*-test and one-way ANOVA with Bonferroni adjusted *post hoc* test, respectively. Contingency tables with Pearson’s chi-squared (χ^2^) test for independence were utilised to assess the categorical variables. Furthermore, to account for variation due to the sample size (unequal sample size between comparators), adjusted residuals were performed to identify which subsets were significant, or contributed the most (residual greater than 1.96; i.e., significantly higher) or the least (residual less than −1.96; i.e., significantly lower) to the relationships, which corresponds to *p* < .05. A *p*-value of <.05 was considered statistically significant. Overall data, as well as differences and/or changes before and during lockdown [i.e., ≥10% (*p* < .05), or otherwise noted] in variables are highlighted in the Results section. Visualization of datasets (figures) were made using GraphPad Prism (8.0.1) and Datawrapper (Datawrapper GmbH, Berlin, Germany).

## Results

Table 1 shows the demographic characteristics of athletes in Malaysia (*n* = 686). Most respondents were male (59%), aged 24.2 ± 7.2 years (18–29 years: 82% of the sample), with competitive experience of 8.1 ± 6.3 years, from 50 sports, mostly athletics (12%). All respondents were of various ethnicities who resided in one of 13 states or 3 Federal Territories in Malaysia (Table 1; Figure 1). During the lockdown, athletes were training at home (74%), with <2% having access to gym facilities. At their homes, para-athletes (43%) received more assistance with training equipment than World-Class (23%), Team (9%) and Endurance (4%) athletes.

Training practices of athletes are shown in Table 2. Athletes trained for general strength and health (88%), and muscular endurance (71%). More International- (62%) and National-level (56%) athletes than recreational athletes (36%) dedicated time to improving muscle balance. More females (42%) than males (29%) sourced training materials from social media (e.g., YouTube). More World-Class (64%) and International (55%) athletes received training programs from coaches, or from coaches combined with their own programs (65% in Internationals). More females (30%) than males (19%) trained with partners of equal capacity (fitness), and similarly for World-Class (42%) compared to State and Recreational athletes (16%). More State athletes were training alone (96%) compared to higher classification-level counterparts (≤86%). As part of lockdown training, more World-Class (48%) than lower classification-level athletes (≤34%) used innovative/modern training modalities (e.g., digital based, Avatars, Zwift racing) to maintain/improve fitness. A greater proportion of higher classification-level (especially World Class athletes, 48%) athletes performed weightlifting/strength training than State athletes (20%). More International (45%) and National (44%) athletes performed plyometric training than the other athlete classifications. Also, more International athletes performed plyometrics (43%) and sport-specific technical skills (46%) than others (Table 2).

Changes in training intensity (Figure 2), frequency, and duration are shown in Figure 3. Overall, the training intensity of sport-specific training during lockdown was approximately 66% of pre-lockdown levels, without a marked difference among the comparators (Figures 2, 3). There was a ∼20% reduction in athletes who trained ≥5 sessions/week, during lockdown. World-Class athletes had similar training frequencies pre- and during-lockdown (84% vs. 85%). Training duration of all athletes was reduced (24%–59%), especially the National (50%), International (39%) and World-Class (40%) athletes. In terms of training space and equipment (Figure 4), athletes had less training space/access (69%), including equipment (69%), for cardiorespiratory training, which was more available for technical and strength training (Figure 4). More World-Class athletes (62%) had greater access to necessary equipment for strength training than the other classifications (<50%) (Figure 4).

Athletes’ monitoring, recovery, and injury prevalence are shown in Table 3; Figure 5. During lockdown*,* contact with a coach/trainer at least once per day occurred more among World-Class (41%) and International (40%) than State (18%) and Recreational (19%) athletes. A large number of World-Class athletes monitored training load (77%) than other athletes. Training monitoring by fitness coaches occurred more commonly among World-Class athletes (45%), than State (19%) and Recreational (7%) athletes. World-Class athletes had their training loads monitored mostly *via* questionnaire (33%) and RPE (27%) scales/methods. One main mode of physical recovery, which was similar across all comparative variables, was stretching (68%). Overall, 38% of athletes included injury prevention exercises (e.g., stretching, stability, mobility, flexibility) and 18% reported experiencing mild injury. Of these, 18% (across all athletes) received a medical diagnosis, mostly among International athletes (31%). The reported injuries (*n* = 82) were mostly related to the knee (29%) and ankle (26%) (Figure 5).

Psychological and financial status of athletes are shown in Table 4; Figure 6. Lower classification athletes, especially State (44%) athletes agreed that the mandated lockdown increased feelings of mental vulnerability. More of these athletes (36%) also agreed feeling anxious during lockdown, compared to higher classification-level athletes (14%–21%). A greater proportion of higher classification-level athletes, especially International (62%) compared to State athletes (42%), developed their mental skills/performance by watching competitions or practising mental training. More Individual-sport (79%) than team-sport (63%) athletes experienced financial difficulty during lockdown, as well as World-Class athletes (86%) compared to National and State athletes (both 67%) (Figure 6).

Sleep pattern data during lockdown is shown in Table 5. Lower-classification athletes practised power naps during the daytime, both before (State 71%, Recreational 78%) and during lockdown (70% and 71%, respectively). More athletes indicated that their sleep quality was “normal” (49%) and improved (35%), than those who reporting “worsened” sleep (16%). Similarly, for sleep quantity, 49% of respondents indicated normal, 27% improved, and 14% worsened. State athletes typically had inferior sleep quality (27%) and quantity (24%) (Table 5).

## Discussion

Key training variables of athletes from Malaysia were substantially altered during the mandated COVID-19 lockdown, especially for different classification athletes (World-Class, National, State, and Recreational). Despite the observed overall reduction in training frequency, the proportion of World-Class athletes training at least five times weekly during lockdown was preserved. This group, and other athletes, however, reduced their training duration per session, training intensity (during sport-specific training), and performed alternative training modes. Most athletes aimed to maintain or develop general strength and health, and muscular endurance, utilising body-weight-based exercises. Interestingly, most of higher classification-level athletes (World-Class, International) received their training programs from coaches, using innovative/modern methods (e.g., digital-based), with more opportunities to perform weightlifting/strength (proper equipment), plyometrics, and sport-specific technical skills than lower classification-level athletes. Higher classification-level athletes had regular contact with coaches/trainers, which facilitated their training monitoring. Even though some athletes practised injury prevention exercises, ∼18% reported suffering from a mild injury (e.g., knees and ankles) during the lockdown. These findings indicate that athletes, coaches, and support staff need logistical support that facilitates training in a lockdown situation.

Athletes reported that lockdown situations made them mentally more vulnerable, along with increased anxiety feeling, especially the lower classification-level athletes, regardless of sport type (individual or team) or gender. Higher classification-level athletes (e.g., International) spent more time training mental skills/performance by watching competitions or undertaking mental training. Interestingly, sleep patterns were generally unaffected as more athletes indicated sleep quality and quantity to be “normal” or “improved” during lockdown compared to pre-lockdown. Conversely, “worsened” sleep was reported among lower classification-level (State) athletes. The majority of athletes reported financial difficulties, especially among individual sports, and World-Class athletes. During lockdown situations, decisive efforts to scale-up assistance (e.g., physical, mental and wellbeing related support) are necessary, e.g., put into place measures that facilitate the training (even if quarantine- or home-based) and protect the wellbeing (including mental and finance related support) of athletes.

Home/modified training (in a lockdown context) appeared to compromise weekly training frequency, intensity of sport-specific training, and session duration. These negative impacts in training variables are likely associated with facility limitations, e.g., limited availability of training space and equipment, as reported among athletes in many other countries worldwide (Pillay et al., 2020; Washif et al., 2022b; Pagaduan et al., 2022). A similar proportion of World-Class athletes (∼86%) maintained their pre- and during-lockdown training frequency of ≥5 times per week. When lockdowns were instigated, many World-Class athletes were already in their preparation for major competitions (e.g., Olympic Games, World cups, and international tours), and endeavoured to practice “everyday”. Some of these athletes also received/bought new equipment, further enabling this training consistency/maintenance (Washif et al., 2022b; Meier et al., 2022). During lockdown, home-based or modified training (i.e., bodyweight-based) widely replaced the traditional resistance training, which is similar to the changes reported in our global study (Washif et al., 2022b). It is reasonable to surmise that these changes were responsible for the 35% reduction in training intensity in the current study. An important caveat is that training intensity is a key component required to preserve fitness (Mujika, 2010), including endurance and strength performance (Izquierdo et al., 2007; Mujika, 2010; McMaster et al., 2013; Spiering et al., 2021). It is important that a decrease in strength level may be observed after a 3-week cessation of resistance training, and exacerbated after ≥5 weeks (McMaster et al., 2013). It would seem that athletes in Malaysia preserved their weekly training frequency (especially the World-Class cohort) when other key training variables (e.g., duration, intensity) were compromised.

Training duration (volume) is crucial among athletes requiring a high level of endurance. We observed that ∼41% of athletes maintained a training duration of ≥60 min (per session), during the lockdown. A similar proportion of athletes training at shorter duration of 30 to ≤60 min (∼43%) or at ≥60 min (46%) during lockdown, globally (Washif et al., 2022b). In this context, a weekly training volume of ∼380 min (4–5 sessions/week) can improve aerobic fitness and maintain muscular power of soccer players (Rampinini et al., 2021a). Essentially, athletes who were in possession of a home treadmill or a bike would be the least affected by the lockdown situations. This group of athletes appeared to preserve the majority of specific training they performed daily (Washif et al., 2022b). In particular, a previous study reported that endurance runners (especially top-level athletes) predominantly undertake low-intensity, long-duration training, with the addition of highly intensive bouts (Seiler, 2010). Meanwhile, a review of 9 studies (in team sports players) described the typical home training comprising of an average ∼5 ± 2 weekly training sessions, with a session duration of ∼45–90 min (Paludo et al., 2022). The latter authors reported VO_2_max changes from +6% to -9% (highly variable results), increased sprint times (.4%–36%) reflective of poorer sprint performance; and changes in countermovement jump height (−5% to +15%) after home/modified training that focused on muscular strength and endurance (Paludo et al., 2022). Furthermore, with similar training focuses, minor changes were reported in reactive agility performance of −12% (Pucsok et al., 2021), and one-repetition maximum strength of −3% (Pedersen et al., 2021). It appears there are highly variable between-athlete changes in physiological and performance changes due to the COVID-19 lockdowns (Paludo et al., 2022).

During the Malaysian lockdown, athletes modified their training aims, and mostly trained for general strength and health, and muscular endurance. It is important that the prevalence of athletes training for muscular endurance was relatively higher (71% vs. 55%) than that reported in a global study (Washif et al., 2022b). These changes were associated with restrictions during the lockdown, further reducing training specificity (e.g., different training modes/types). It is important that, unlike the “transition phase,” “altered training” during lockdown is usually designed to limit detraining effects i.e., to maintain or even improve performance (Washif et al., 2022c). This approach can be facilitated by regular contact with coaches/trainers, i.e., training monitoring (e.g., loading regulation) and prescriptions (e.g., training programs); for athletes to perform an appropriate training program that permits adaptation while reducing the risk of “overreaching” and injury. In the current study, such provisions were seen more often among higher classification-level athletes (i.e., World-Class, International). According to a recent study, World-Class athletes were the least to adopt a self-designed training routine, since they received the greatest support from their coaching team (Pagaduan et al., 2022). Indeed, a structured home-based training program during the COVID-19 lockdown preserved lower limb explosive strength (Font et al., 2021). Additionally, a 3-month period of home-based and group-based interventions (mainly for strength, jump, and sprints) during lockdown was effective for maintaining strength, jumping, and sprinting ability among high-level female football players (Pedersen et al., 2021). In the case of facing limited resources (lockdown situation), a tailor-made home-based exercise program (typically bodyweight-based; Yousfi et al., 2020) not only ensures regular and consistent training, but helps promote health-related benefits (mental, emotional, and physical). Further, athletes may take advantage of the hot and humid conditions (e.g., Malaysia), and use clothing/garments that restrict heat loss while training at home as a means to ‘heat up’ the body temperature and heart rate to boost metabolic responses (Willmott et al., 2018). Even though beneficial for fitness maintenance (or improvement), the safety ramifications of home-based and modified training have not been assured (Washif et al., 2022b). In the current study, ∼18% of athletes reported a mild injury, mostly knee and ankle injuries, irrespective of sports, gender, and level of athletes, despite ∼36% of the athletes implementing injury prevention exercises as part of their training at home. These phenomena could be as a result of the abrupt resumption of high-intensity training to be ready for the competitions after lockdown (Bazett-Jones et al., 2020; DeJong et al., 2021).

Remote conditioning training aims at maintaining some level of training and preserving some social connections. Collaterally, decreases in training were linked with higher depression, anxiety, and stress symptoms during the COVID-19 lockdown (Facer-Childs, et al., 2021). Some elite athletes reported psychological distress due to training cessation during lockdown (di Cagno et al., 2020; Jia et al., 2022; Venturelli et al., 2022), changes in sleep (Facer-Childs et al., 2021; Romdhani et al., 2022b), financial problems, among others. In the current study, the majority of athletes reported financial difficulties, especially among individual-sport, and World-Class athletes; an extra stressor that potentially impacted the athletes’ mental health (Busch et al., 2022). Individual and team-based sports obtained mixed results with both categories indicating increased risks for poor mental and emotional health (Jia et al., 2022). In professional football, the prevalence of anxiety (8%–18% in females) and depression (6%–13% in males) increased during the COVID-19 lockdown (Gouttebarge et al., 2022). Athletes in the present study, especially the lower classification-levels, reported that they felt mentally vulnerable, and similarly, had increased anxiety levels. Such observations may relate to reduced or missing interactions with teammates, including “online team training” that was also limited among lower classification-level athletes, as shown in the current study. In contrast, higher classification-level athletes might have adequate (more) social support and/or connections (e.g., during “online training” sessions), with some athletes practising their mental skills to improve their mental status. Concomitantly, mental health was associated with lockdown-induced sleep pattern changes (i.e., increased total sleep time and sleep latency) (Facer-Childs et al., 2021). In the current study, sleep patterns were generally unaffected as more athletes indicated that their sleep quality and quantity were “normal”, and for some sleep was “improved” during the lockdown. However, relatively poorer sleep patterns were reported among lower classification-level (State) athletes. We are unable to directly identify what caused the worsening of sleep in the lower classification-level athletes, but it can likely be attributed to several factors, including financial difficulties and/or reduced social connections during training.

To our knowledge, only a few country-specific studies have investigated training practices, recovery, sleep, mental, and injury prevalence of athletes for different classification athletes (recreational to World-Class levels), different sports, and gender. Here we report the results of athletes from 50 sports (individual and team). However, this study is not without limitations. Subjective questionnaires were used to obtain responses from participants retrospectively and therefore subject to recall bias. Here we considered a convenience sampling approach that limits the generalisability of our findings. Furthermore, a qualitative analysis through interviews might address more detailed (or specific) issues of athletes from a different perspective of performance; whereas questionnaires we used were specific to our objectives, albeit checked and verified by a large number of researchers and scientists including experts (e.g., in sports performance, periodisation, detraining, recovery, sleep, psychology, injury, among others). Ramadan fasting occurred in the early the lockdown, and might have impacted on the results (Romdhani et al., 2022a), which we addressed elsewhere (Washif et al., 2022d). Moreover, data concerning injury prevalence is only specific to athletes with acute mild (light) injury as we did not consider the participation (survey) of athletes having moderate or severe injuries, which further limits the conclusions regarding injury prevalence during the lockdown.

## Conclusion

The early mandated COVID-19 lockdown in Malaysia (2020) induced more severe effects on the training-related practices of lower classification-level athletes. World-Class athletes experienced the fewest effects on training-related issues (e.g., training programs, sport-specific training, monitoring, and equipment). Overall, the key training variables of frequency, intensity, duration, and modes were compromised. During the lockdown, athletes focused more on preserving (or possibly enhancing) general strength and health, with an emphasis on muscular endurance given high accessibility to bodyweight-based related exercises. Some other exercises (e.g., plyometrics) were emphasised and their use was mediated by athlete classification level. Training at home (including modified training) was associated with safety considerations, as a substantial proportion of athletes reported having had mild “injuries” during the lockdown. Recognising the associated challenges, a one-size-fits-all program may not be ideal during lockdowns. Thus, we recommend objective-driven country-specific support to athletes; possibly implemented as home-based support e.g., fitness training, psychology, financial, and lifestyle. It might be necessary to consider an arrangement that permits “bubble training/competition” to reduce lockdown-associated challenges, and facilitate regular training during the lockdown, not only for high classification-level athletes, but also State- and/or National-level athletes.

## Data Availability

The original contributions presented in the study are included in the article/Supplementary Material, further inquiries can be directed to the corresponding author.
